# p62 links the autophagy pathway and the ubiqutin–proteasome system upon ubiquitinated protein degradation

**DOI:** 10.1186/s11658-016-0031-z

**Published:** 2016-12-13

**Authors:** Wei Jing Liu, Lin Ye, Wei Fang Huang, Lin Jie Guo, Zi Gan Xu, Hong Luan Wu, Chen Yang, Hua Feng Liu

**Affiliations:** 1 The Institute of Nephrology, Guangdong Medical University, Zhanjiang, Guangdong 524001 China; 2 Key Laboratory of Chinese Internal Medicine of Ministry of Education and Beijing, Dongzhimen Hospital Affiliated to Beijing University of Chinese Medicine, Beijing, 100700 China

**Keywords:** p62, Autophagy, Ubiquitin–proteasome system (UPS), Ubiquitinated protein, Aggresome, Proteostasis, p62 phosphorylation, Keap1–Nrf2 pathway, Histone deacetylase 6 (HDAC6), Mechanistic target of rapamycin complex 1 (mTORC1)

## Abstract

The ubiquitin–proteasome system (UPS) and autophagy are two distinct and interacting proteolytic systems. They play critical roles in cell survival under normal conditions and during stress. An increasing body of evidence indicates that ubiquitinated cargoes are important markers of degradation. p62, a classical receptor of autophagy, is a multifunctional protein located throughout the cell and involved in many signal transduction pathways, including the Keap1–Nrf2 pathway. It is involved in the proteasomal degradation of ubiquitinated proteins. When the cellular p62 level is manipulated, the quantity and location pattern of ubiquitinated proteins change with a considerable impact on cell survival. Altered p62 levels can even lead to some diseases. The proteotoxic stress imposed by proteasome inhibition can activate autophagy through p62 phosphorylation. A deficiency in autophagy may compromise the ubiquitin–proteasome system, since overabundant p62 delays delivery of the proteasomal substrate to the proteasome despite proteasomal catalytic activity being unchanged. In addition, p62 and the proteasome can modulate the activity of HDAC6 deacetylase, thus influencing the autophagic degradation.

## Introduction

Nearly 30% of newly synthesized proteins in the cell are misfolded under normal conditions [1]. Two systems that maintain cellular proteostasis are the ubiquitin–proteasome system (UPS) and autophagy. These self-governed systems degrade various substrates, and while they are distinct, a growing body of evidence indicates cooperation between them. They share some ubiquitinated proteins, such as HttQ74, a huntingtin protein in Huntington’s disease [2], but also degradation elements, such as p62.

**Fig. 1:**
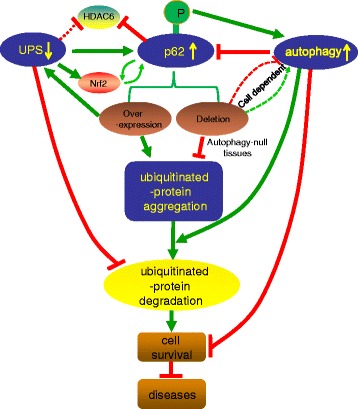
The interactions of p62 and the UPS, autophagy and ubiquitinated proteins. Upon UPS inhibition, p62 is upregulated and phosphorylated on S405 and S409, which can facilitate the degradation of ubiquitinated cargoes via autophagy. p62 synthesis is induced by an increase in Nrf2 following UPS deficiency. The increased p62 competes with Nrf2 for Keap1, and then a p62–Keap1 complex selectively facilitates the ubiquitinated aggregate formation and creates a positive feedback loop with Nrf2. HDAC6 can be activated by the products in UPS (such as K63), but inhibited directly by p62. HDAC6 plays a critical role in ubiquitinated aggregate formation and autophagosome–lysosome fusion, while a ratio of p62 to HDAC6 maintains the homeostasis of autophagic process. Besides inhibiting the degradation of p62 and ubiquitinated proteins, a deficiency in autophagy also compromises UPS since the increased p62 delays ubiquitinated protein delivery to UPS for degradation. p62 overexpression increases the aggregation of ubiquitinated proteins and has a protective effect on cell survival, while p62 deletion exacerbates cell injury and relates to some diseases by either facilitating or damaging autophagic degradation dependent on the cell type

p62 is an autophagy substrate that is used as a reporter of autophagy activity. Recently, p62 was also shown to deliver ubiquitinated proteins, such as tau, to the proteasome for degradation. In addition, it can shuttle between the nucleus and cytoplasm to bind with ubiquitinated cargoes and facilitate nuclear and cytosolic protein quality control. Other functions of p62 are gradually being revealed, emphasizing its importance in the proteolytic system. This review focuses on the role of p62 in linking the ubiquitin–proteasome system and autophagy pathway upon ubiquitinated protein degradation (Fig. 1).

## Ubiquitin–proteasome system

**Fig. 2:**
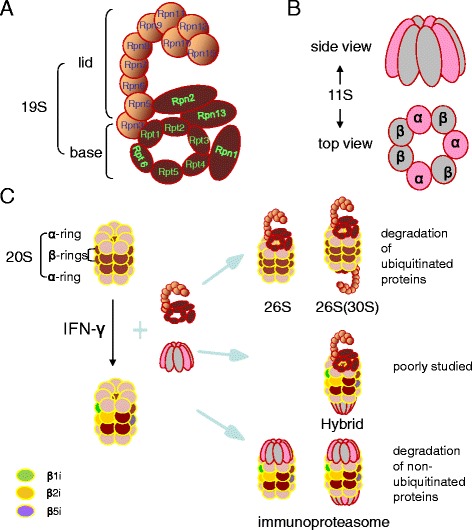
Structures of the mammalian proteasome. **a** A simplified model of the proteasome regulatory particle 19S. The lid mainly de-ubiquitylates the captured substrates, while the base functions as substrate unfolding and translocation. Rpn11 serves as a de-ubiquitylating enzyme (DUB) *en clon* cleaving the polyubiquitin chain of substrates. Rpt1-6, an ATPase ring, is involved in substrate protein unfolding and translocation into the channel of the 20S. Rpn13 and Rpn10 (a lid subunit) serve as ubiquitin receptors. Rpn1 can bind to the ubiquitin shuttle receptors and cytoplasmic deubiquitinases. **b** A simplified model of the proteasome regulatory particle 11S, which is also termed PA28. It is an activator of the proteasome. **c** Assembly model of mammalian proteasome. 20S binding 19S at one or two ends generates the 26S proteasome (or 30S), with an ATP-dependent degradation of ubiquitinated substrates. Upon stimulation of interferon-γ (INF-γ), all three active subunits (β1, β2 and β5) of the constitutive 20S proteasome are replaced by close-proximity similar subunits (β1i, β2i and β5i, respectively) that bind to 11S to generate the immunoproteasome. The immunoproteasome responds to antigen presentation with a non-ATP-dependent degradation of non-ubiquitinated proteins

The ubiquitin–proteasome system (UPS) plays a critical role in the degradation of short-lived, misfolded and damaged proteins. This is necessary to maintain protein homeostasis, cell cycle control [3], inflammation, oxidative stress, apoptosis [4] and immunity [1]. It even serves a non-proteolytic function in the control of translation [5]. The proteasome is a highly conserved protease complex consisting of two moieties that combine into a diversity of forms: the 20S catalytic core particle and the 19S or 11S regulatory particle(s) (Fig. 2). 20S is a barrel-shaped complex that possesses two α-rings and two β-rings, with each ring composed of seven subunits. β1, β2 and β5 are 3 subunits of each β-ring, respectively possessing peptidylglutamyl peptide-hydrolyzing or caspase-like activity (PGPH or C-L); trypsin-like activity (T-L); and chymotrypsin-like activity (CT-L) [6]. 19S consists of a lid and a base, which is involved in substrate recognition, deubiquitination, unfolding and further translation into 20S for degradation [7, 8].

The progress of proteolysis also requires ubiquitin to covalently attach to substrates. This 76-amino acid protein can form an isopeptide bond between its C-terminal glycine (G76) and a lysine residue within the target molecules or ubiquitin itself [9]. Ubiquitination is completed via an enzymatic cascade involving E1 ubiquitin-activating enzyme(s), E2 ubiquitin-conjugating enzyme(s) and E3 ubiquitin ligase(s). Then the ubiquitinated proteins are recognized and degraded by the 26S proteasome, which consists of a 20S unit with one or two 19S units at one or both ends [10].

## Autophagy

Autophagy is a highly evolutionarily conserved degradation system in eukaryotes [11]. It was first considered to be a non-selective bulk system for degrading long-lived proteins and organelles to recycle nutrients and generate energy [12]. Later studies showed that autophagy selectively degrades protein aggregates (aggrephagy), peroxisomes (pexophagy), damaged mitochondria (mitophagy), intracellular bacteria and viruses (xenophagy), surplus endoplasmic reticulum (reticulophagy), ribosomes (ribophagy) and mid-body ring structures [13].

The autophagic degradation model of eukaryotes is emerging through more recent research [12]. Autophagy begins with the formation of a phagophore, which is a crescent-shaped double membrane tightly associated with LC3II. The phagophore engulfs adaptor-mediated ubiquitinated substrates to become an autophagosome, which fuses with the lysosome to become an autolysosome with an internal acidic, hydrolytic environment that helps to degrade the content [14]. For content outside the cell, the cell membrane caves to envelop it. This is an endosome, which fuses with an autophagosome to become an amphisome, which in turn fuses with a lysosome to become an autolysosome.

Autophagy-related gene (Atg) proteins play essential roles in autophagy. They are known as the ‘core machinery’ [15]. More than 40 Atg proteins have been identified as participating in autophagy or autophagy-related processes [16]. p62 and NBR1 (neighbor of BRCA1 gene 1) are two important cargo receptors involved in selective autophagy. They are essential in the formation of ubiquitinated aggregates [17, 18]. NBR1 is twice as large as p62, has a similar domain architecture, and shares several key features with it. Cellular NBR1 is modulated by the autophagic process and does not seem to be influenced by proteasomal degradation [17]. Recent studies have revealed a critical role for autophagy in some human diseases, such as tumors [19], neurodegenerative diseases and aging. Some cell lines, such as podocytes, have high basal autophagy [20].

## p62

p62 was the first selected autophagy adaptor discovered in mammals [11, 21, 22]. It was termed sequestosome 1 (SQSTM 1) by Shin due to its ability to form aggregates [23]. A170 and ZIP are the respective names for the mouse and rat variants.

**Fig. 3:**
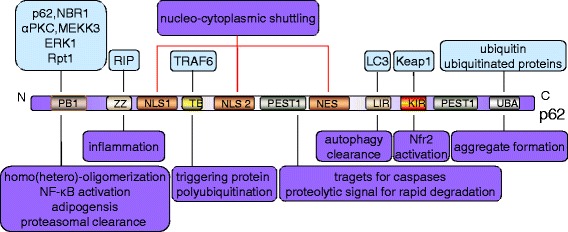
Structure and function of p62. The *light blue block charts* represent the interacting proteins and the *light purple* ones represent the function. p62 can assemble via the N-terminal PB1 domain (Phox and Bem1) with itself or with NBR1, termed homo- or hetero-oligomerization, respectively. The PB1 domain also interacts with atypical PKC (αPKC) and MEKK3, accounting for NF-κB activation with ERK1 and Rpt1 for adipogenesis and proteasomal clearance, respectively. The ZZ domain binds RIP1 kinase, which is responsible for inflammation. The TRAF6-binding domain (TB) interacts with TRAF6 proteins to trigger protein polyubiquitination. The nuclear localization signal (NLS1/2) and the export motif (NES) are involved in the nucleo-cytoplasmic shuttling of p62. PEST1 serves as a proteolytic signal for rapid degradation relevant to short-lived proteins and as targets for caspases. Through the LIR and the UBA, p62 assists in the autophagic degradation of ubiquitinated proteins. KIR binding to Keap1 leads to Nrf2 activation

p62 is a multifunctional protein consisting of an N-terminal Phox-BEM1 domain (PB1), a ZZ-type zinc finger domain, a nuclear localization signal (NLS), an export motif (NES), an LC3-interacting region (LIR), a Keap1-interacting region (KIR), and a C-terminal ubiquitin-associated domain (UBA) [24, 25] (Fig. 3). p62 interacts non-covalently with ubiquitin or polyubiquitin chains via the UBA domain, and then delivers polyubiquitinated cargoes to autophagy via the LIR domain (which is also known as the Atg8 family-interacting motif), and to the proteasome via the PB1 domain [23, 26]. In addition to a high potential for homo-oligomerization [27, 28], the PB1 domain can also hetero-oligomerize with NBR1 or other PB1 domain-containing proteins, such as atypical protein kinases Cs (αPKCs), MEKK3, MEK5, ERK1 and Rpt1, which modulate different signaling pathways and get involved in osteoclastogenesis, angiogenesis and early cardiovascular development or cell polarity [29].

The oligomerization of p62 via the PB1 domain is critical for ubiquitinated protein accumulation in autophagy-null cells [30]. αPKCs and MEKK3 can activate NF-κB signaling by binding the PB1 domain of p62, respectively with the assistance of the receptor-interacting protein-1-binding (RIP-binding) ZZ domain and tumor necrosis factor receptor-associated factor 6-binding (TRAF6-binding) TB domain [31, 32]. The p62–TRAF6 complex appears to modulate the ubiquitination of the IKK complex [33]. The specific role of MEK5 binding to p62 is actually unclear at present. p62 controls adipogenesis and obesity via interaction with ERK1 [34]. The functions of the other domains will be covered in later chapters.

The intracellular level of p62 is dependent on transcriptional regulation and post-translational autophagic degradation. The transcription of p62 is modulated by oxidative stress (Nrf2), the Ras/MAPK pathway, the JNK/c-Jun pathway and some chemical compounds (e.g., resveratrol, an inducer of autophagy) [35]. Proteasome inhibition and starvation can also induce p62 synthesis [36]. On the other hand, p62 is modulated by autophagy since it acts as a substrate during autophagic degradation.

## Ubiquitinated protein degradation pathway

Ubiquitination is integral to the proteolytic system. Ubiquitin possesses 76 amino acid residues and is highly conserved from yeast to humans. It also possesses some non-proteolytic functions, including vesicle trafficking via ubiquitination of membrane proteins, protein kinase activation, DNA repair and chromatin dynamics through monoubiquitinated histone. A common mechanism involves ubiquitin or polyubiquitin chains recruiting ubiquitin receptors to perform biological functions [37].

Ubiquitin is involved in three degradation pathways (UPS, lysosome and autophagy). The triage of ubiquitinated proteins is probably based on their location, the ubiquitin chain length and the linkage types. The three degradation pathways may be interdependent on the ubiquitin pool in the cell [38]. Based on the lysine residues inside ubiquitin, seven homogeneous polymer chain linkages can be defined: K6, K11, K27, K29, K33, K48 and K63 [39].

The K48 chain is a classical sign of proteasomal degradation [39]. K63 is a common marker in the autophagy process [40]. However, recent studies revealed that the proteasome seems to accept almost all types of ubiquitin chain, including homogenous, heterogeneous, linear, head-to-tail, single and multi-branched chains and even those with mono- or multi-monoubiquitination [39, 41]. It is believed that ubiquitin polymer chains consist of at least four ubiquitin moieties [38]. Autophagy can also accept many types of ubiquitin chain [42].

It should be noted that K48 is still the common linkage targeting proteins to the proteasome. During proteasomal degradation, the length of the substrate proteins determines whether the process is mediated by mono- or polyubiquitination [43]. The existence of linkage multiplicity implies subtle alterations that contribute to the strength and/or conformation of the interaction between the proteasome and the substrates. Subtle alterations may control their “priority” to the proteasome, resulting in altered degradation rates that give rise to multiple biological functions [39].

The UBA domain of p62 can bind K48 and K63 (with a higher affinity for K63) [40, 44, 45]. UBA phosphorylation may enhance the affinity for K48 and K63 [46]. The longer ubiquitin chains show higher affinity for p62 [23]. K11 and K13 are thought to have non-proteolytic functions [47], and the other polyubiquitin chains have functions that remain unclear.

Determining the overall contribution of UPS and autophagy to ubiquitinated protein degradation is a topic of considerable interest. Studies with subjects ranging from *Drosophila* to mice suggest that the inactivation of autophagy by pharmacological or genetic inhibition causes the accumulation of ubiquitinated proteins in the brain [48, 49], skeletal muscle, cardiac muscle, pancreatic β-cells, podocytes and hepatocytes. However, Natura et al. obtained the opposite results [26]. In their study, they compared the turnover dynamics of endogenous ubiquitinated proteins by proteasome and autophagy by assessing the effects of their inhibitors. They found that pharmacological or genetic inhibition of autophagy could not increase the ubiquitinated protein level, although proteasome inhibition by epoximycin did. However, many proteins are degraded by both UPS and autophagy [2, 50]. Different cell lines may account for this discrepancy.

## The role of p62 in autophagy

### The relationship between p62 and autophagy

As mentioned previously, p62 delivers ubiquitinated cargoes for autophagic degradation via the C-terminal UBA domain or the LIR domain, and then the PB1 domain promotes the process [26]. Activating autophagy reduces the expression of p62. Pharmacological and genetic inhibition of autophagy can increase the level of p62 in various cell lines (e.g., HeLa cells [22] and neonatal rat ventricular myocytes [51]), tissues (e.g., cardiomyocytes [51], pancreatic β-cells [52], skeletal muscle [53], liver [54] and central nervous system [48]), and species (e.g., mice [11] and *Drosophila* [55]).

In HeLa cells, p62 overexpression has no influence on the autophagic vacuole number and p62 knockdown does not have an effect on autophagic flux [2], indicating that p62 is not required for autophagosome formation under basal condition and starvation. However, in some cell lines, overexpressed p62 enhances protein aggregation and has a protective effect on cell survival [22, 56, 57]. Moreover, p62 deletion results in the impaired formation of the LC3-II, aggresome and autophagosome, exacerbating cell injury and lowering cell viability under basal conditions and misfolded protein stress in cardiomyocytes [58]. On the other hand, silencing p62 can also activate autophagy, as evidenced by an increase in the conversion rate of LC3I to LC3II and in the amount of multilayered autophagosomes (which may present mis-regulated autophagy) in several carcinoma cell lines. Autophagic cell death is the result [59]. Thus, although the impact of p62 on autophagy is cell-dependent, p62 deletion leads to cell death in almost all of the cell lines.

p62 is localized to ubiquitin-positive inclusions, which is a common phenomenon that can be observed in some diseases, such as neurodegeneration [60]. Collectively, p62 relates to the formation of ubiquitin-positive inclusions and binds LC3II to facilitate autophagic degradation. Some studies demonstrated that a reduced p62 level is accompanied by reduced formation of ubiquitin-positive aggregates in autophagy-null mice, but not in autophagy-normal mice [11]. A similar result was found in *Drosophila* [55]. The total amount of ubiquitinated protein in p62-knockout tissues is less than that found in autophagy-null tissues [11]. Therefore, p62 is critical for the aggregation of ubiquitinated proteins [11, 21, 55]. In addition, there may be other pathways that form ubiquitinated aggregates [2].

### p62 as an autophagic flux reporter

p62 is widely used as a predictor of autophagic flux [2, 61], since it is a thoroughly explored autophagic substrate. However, many factors should be considered when assessing autophagic activity using p62. First, p62 interacts with several signaling molecules, which affects its transcriptional synthesis, increasing the complexity of p62 as an autophagic flux reporter. For example, the KIR domain of p62 binds Keap1 to free Nrf2, which can then induce p62 synthesis [62]. In addition, p62 is a key sensor of the mTOR pathway, in which p62 is induced by amino acid shortage [63]. On the other hand, some agents, such as the phosphatidylinositol 3-kinase (PtdIns3K) inhibitor LY294002, actinomycin D and wortmannin, can inhibit p62 protein synthesis [35, 64].

Another issue is the degradation process. It has been reported that Pan-cathepsin inhibitors and bafilomycin A1 can stop the degradation of p62 [35]. We should also take UPS into account, since the p62 protein level increases upon proteasome inhibition, as described above. Finally, overexpressed p62 (e.g., p62-GFP) tends to self-aggregate, which manifests as an inaccurate reduction in autophagy activity [65]. Therefore, when assessing autophagy flux with p62 analysis, it is advisable to use other assessments as well, such as the mRNA level of p62 and LC3-II turnover.

During starvation, the expression level of p62 does not always inversely correlate with autophagy activity. Not only can autophagy be induced, but p62 transcriptional synthesis is also active upon starvation [63, 66]. p62 is restored to basal levels upon prolonged starvation via transcription upregulation triggered by amino acid shortage, although p62 is reduced by autophagic degradation during the early hours [66].

The significance of p62 restoration might be the integration of different proteins to fulfill specific functions, since the other domains of p62 get involved in many signal pathways. Besides, p62 knockdown likely activates autophagy through mTORC1 inhibition in response to starvation, since p62 is a positive regulator of mTORC1 [63]. This creates a feed-forward loop in which mTORC1 activation increases p62 levels, further promoting mTORC1 activity.

## A role for p62 in the UPS

Natura et al. used the proximity ligation assay (PLA) to reveal that p62 and the proteasome are co-localized in situ under basal conditions. They also found that p62 aggregates contain inactive proteasome, ubiquitinated proteins and autophagosome upon proteasome inhibition [26]. It has been shown that p62 can shuttle K63-polyubiquitinated tau for proteasomal degradation [67]. This leads us to explore the relationship between p62 and proteasome.

The N-terminal PB1 domain of p62 might interact with Rpt1 and S5a/Rpn10 of the 26S proteasome and collaborate with the C-terminal UBA domain of p62 by binding ubiquitinated proteins to facilitate UPS degradation [26, 66, 68]. p62 continuously undergoes rapid nucleo-cytoplasmic shuttling using its own two nuclear localization signal domains (NLS1 and NLS2) and one nuclear export motif (NES) [63]. p62 is localized in nuclear aggregates [69] and plays a critical role in recruiting the proteasome to the ubiquitinated inclusion in the nucleus. It may also export ubiquitinated cargoes from the nucleus to the cytosol for more efficient degradation [68].

These studies indicate that p62 is also involved in the proteasomal degradation of ubiquitinated proteins in the nucleus via its NLS and NES domains and in the cytosol via its PB1 domain. Therefore, it naturally contributes to both nuclear and cytosolic protein quality control. Besides, the PEST domain serves as a proteolytic signal for rapid degradation, leading to short intracellular half-lives, which may relate to proteasome function [26]. For instance, HS-1-associated protein X-1 (Hax-1) undergoes a fast turnover via the proteasome system through its PEST domain [70].

Pharmacological inhibition of UPS enhances p62 transcription [26, 71] and induces the accumulation of ubiquitinated proteins. Inhibiting the proteasome with epoximycin increases the level of p62 far beyond the levels induced by autophagy inhibitors [26]. When p62 is overexpressed, proteasome catalytic activity will be not influenced, although UPS substrates accumulate [2], implying that p62 delays the delivery of ubiquitinated proteins to the UPS for degradation. Moreover, p62 overexpression along with pharmacological inhibition of UPS and/or autophagy does not further increase ubiquitin aggregates. These studies suggest that p62 is not required for all of the ubiquitinated aggregates.

## Relationship of p62 with ups and autophagy

### Interdependence upon defective proteostasis

Overexpressed p62 can enhance protein aggregation and has a protective effect on cell survival as described above. p62 deletion barely decreases the amount of ubiquitinated puncta in autophagy normal cells. Although p62 is not necessary for all the formation of ubiquitinated aggregation, it still plays a crucial role in aggregate degradation.

Proteasome inhibition can activate autophagy, in which p62 is the bridge [72]. First, proteotoxic stress imposed by proteasome inhibition can induce p62 phosphorylation at serine 405 (S405 in the UBA domain, which is equivalent to S403 in human) and S409 through ULK1/Atg1, which modulates its binding to ubiquitinated proteins [73]. This increased affinity can stabilize ubiquitinated proteins in the sequestosome, which, in turn, prevents p62 dephosphorylation and leads to efficient degradation of the protein aggregates [74]. S409 phosphorylation is essential for the autophagic degradation of ubiquitinated proteins, recruitment of autophagy machinery proteins and facilitation of S405 phosphorylation by ULK1, casein kinase 2 (CK2) [29] or TBK-1 [46]. Furthermore, proteasome deficiency upregulates p62 transcription [36]. Finally, proteasome deficiency can induce adaptive transcriptional activation of Nrf2, which can induce p62 synthesis [35].

The S351 of KIR is phosphorylated, leading to a rising affinity of p62 for Keap1 and followed by sequestration of Keap1 on the cargoes [75]. Subsequently, Nrf2 is stabilized and shuttled into the nucleus to function. The p62–Keap1 complex selectively facilitates aggregate formation entrapped by autophagosomes [76] and creates a positive feedback loop with Nrf2 [62, 77].

NF-E2-related factor 2 (Nrf2), a transcription factor that controls the expression of an abundance of anti-oxidant genes, is degraded by the proteasome via the Cul3–Keap1–E3 ligase complex under basal conditions. However, during oxidative stress, p62 expression is upregulated by the nuclear import of Nrf2 resulting from the blocked interaction between Keap1 (a negative regulator of Nrf2) and Nrf2. The increased p62 can compete with Nrf2 for Keap1 at the Nrf2-binding site, forming a positive feedback loop [62, 78]. All of the investigations suggest that cellular defense mechanisms are networked to fight against defective proteostasis and p62 is a center regulator.

While proteasome deficiency enhances autophagy, autophagy inactivation compromises the ubiquitin–proteasome system due to surplus p62, which delays proteasomal substrate delivery to the proteasome with no changes shown in proteasomal catalytic activity [2]. There is another opinion that pharmacological or genetic inhibition of autophagy can activate the proteasome, as evidenced by an increase in proteasomal activities and the upregulation of proteasomal subunits under nutrient-deficient conditions [79]. It is plausible that the situation occurring in the cell upon starvation is much different from other conditions, such as in the state of overabundant proteins. For instance, p62 can no longer reflect autophagic activity during starvation as mentioned above. Phosphorylation of p62 by ULK1 may enhance autophagic clearance, but that does not occur during starvation, despite its role in canonical autophagy signaling [73].

## Cooperation in aggresome degradation

The terms ‘aggresome’, ‘aggregate’, and ‘inclusion bodies’ are used to describe misfolded protein granules in cells. In 1998, the aggresome was defined as a ‘pericentriolar membrane-free, cytoplasmic inclusion containing misfolded ubiquitinated proteins encased in a cage of intermediate filament proteins that co-localize with the microtubule organizing center (MTOC)’, in which the autophagosome and lysosome fuse [80]. The ‘aggregate protein’ can be concentrated to become the aggresome from the periphery to the peri-nucleus. The ‘inclusion bodies’ have a broader definition that is not microtubule dependent [81]. This terminology is challenged because it gives the impression of a static state, while many of the bodies are actually highly dynamic and reversible. The term ‘dynamic droplets’ was suggested to describe the dynamic liquid-phase structures as opposed to the solid-phase structures (amyloid-like aggregates) [82]. Further investigation is required to make a final affirmative determination.

Although the aggresome was initially used to describe the disease-associated inclusion bodies formed in neurodegenerative diseases, e.g., Lewy bodies in Parkinson’s disease and hyaline inclusion bodies in amyotrophic lateral sclerosis (ALS), the relevance of the aggresome to inclusions in disease is still disputable [77]. Almost a decade ago, disease-related proteins, such as huntingtin (Htt), were found to form a different pattern compared with the misfolded protein upon proteasome inhibition [83], i.e., periphery versus peri-nuclear, despite other similar biological characteristics.

There is a common consensus that misfolded proteins aggregate and are concentrated in the aggresome, which is removed via the autophagy–lysosome pathway [77]. Degradation is strongly based on the activity of histone deacetylase 6 (HDAC6), which also plays a pivotal role in aggresome formation [84]. Acetylated cortactin becomes cortactin via HDCA6 deacetylase activity, and the latter interacts with F-actin to form cortactin–F-actin assemblies that are recruited to the MTOC, promoting autophagosome and lysosome fusion and substrate clearance. p62 can modulate this process by directly inhibiting HDAC6 activity and facilitating removal of the cortactin–F-actin assembly to MTOC, which seems paradoxical [84]. Some observations suggest that loss of p62 leads to cortactin–F-actin assemblies remaining localized in the periphery and ubiquitinated protein accumulation [85]. HDAC6 knockdown leads to failure of fusion between the autophagosome and lysosome and subsequent protein aggregation [86]. p62 can facilitate protein aggregation and also modulate protein transport to the processing site, while HDAC6 facilitates autophagosome–lysosome fusion. The ratio of p62 to HDAC6 maintains the homeostasis of the autophagic process. The proteasome can also modulate aggresome degradation: Poh1, a subunit of 19S, cleaves ubiquitin chains from the substrates, and subsequently the products and K63 activate HDAC6 [87]. While proteasome inhibition imposes proteotoxic stress, the cell fate (survival or death) in response to an altered ratio of p62 to HDAC6 remains unclear.

## Conclusion

Because the UPS, autophagy and p62 are the interdependent elements of the protein quality control system, they must act in a networked manner to maintain proteostasis. p62 may serve as an integration center for multiple functions, including the formation of the autophagosome, the delivery of ubiquitinated proteins to the proteasome, and aggregate formation for autophagic clearance. It is also involved in several signaling pathways [88, 89]. In addition, it has been shown that p62 can inhibit ATP- and ubiquitin-independent LC3 degradation by the proteasome [90]. p62 is also involved in many diseases. For instance, the mutation of the UBA domain in p62 leads to Paget’s disease [91]. p62 and autophagy synergize to promote tumor growth [92], and p62 selectively binds mutant SOD1 to form aggregates in model systems of familial amyotrophic lateral sclerosis [25, 57]. Therefore, p62 could be a promising strategic target for treatment of certain pathological conditions.

## Abbreviations

HDAC6: Histone deacetylase 6
MTOC: The microtubule organizing center
mTORC1: Mechanistic target of rapamycin complex 1
Nrf2: NF-E2-related factor 2
UPS: The ubiquitin–proteasome system
αPKCs: Atypical protein kinases Cs
